# The effect of abscisic acid chronic treatment on neuroinflammatory markers and memory in a rat model of high-fat diet induced neuroinflammation

**DOI:** 10.1186/s12986-016-0137-3

**Published:** 2016-10-26

**Authors:** Sandra Sánchez-Sarasúa, Salma Moustafa, Álvaro García-Avilés, María Fernanda López-Climent, Aurelio Gómez-Cadenas, Francisco E. Olucha-Bordonau, Ana M. Sánchez-Pérez

**Affiliations:** 1 Department of Medicine, University of Jaume I, Vicente Sos Banyat s/n, 12071 Castellón de la Plana, Castellón Spain; 2 Department of Agriculture and Environmental Sciences, University of Jaume I, Vicente Sos Banyat s/n, 12071 Castellón de la Plana, Castellón Spain

**Keywords:** Obesity, Working memory, Microglia, Phytohormones

## Abstract

**Background:**

Western diet and lifestyle are associated with overweight, obesity, and type 2 diabetes, which, in turn, are correlated with neuroinflammation processes. Exercise and a healthy diet are important in the prevention of these disorders. However, molecules inhibiting neuroinflammation might also be efficacious in the prevention and/or treatment of neurological disorders of inflammatory etiology. The abscisic acid (ABA) is a phytohormone involved in hydric-stress responses. This compound is not only found in plants but also in other organisms, including mammals. In rodents, ABA can play a beneficial role in the regulation of peripheral immune response and insulin action. Thus, we hypothesized that chronic ABA administration might exert a protective effect in a model of neuroinflammation induced by high-fat diet (HFD).

**Methods:**

Male Wistar rats were fed with standard diet or HFD with or without ABA in the drinking water for 12 weeks. Glucose tolerance test and behavioral paradigms were performed to evaluate the peripheral and central effects of treatments. One-Way ANOVA was performed analyzed statistical differences between groups.

**Results:**

The HFD induced insulin resistance peripherally and increased the levels of proinflammatory markers in in the brain. We observed that ABA restored glucose tolerance in HFD-fed rats, as expected. In addition, chronic ABA treatment rescued cognitive performance in these animals, while not affecting control diet fed animals. Moreover, it counteracted the changes induced by HFD in the hypothalamus; microglia activations and TNFα mRNA levels.

**Conclusion:**

These results suggest that ABA might become a new therapeutic molecule improving the neuroinflammatory status and insulin resistance.

## Background

Obesity, a leading cause of type 2 diabetes [1], correlates with cognitive impairment. Insulin sensitizers have been proposed as a promising tool for the reduction of obesity-induced insulin resistance and inflammation processes. The thiazolidinediones (TDZ) are a family of synthetic insulin sensitizer molecules; however, some of them have undesirable side effects [2–4]. Thus, alternative compounds with analogous properties but fewer side effects are needed. The phytohormone abscisic acid (ABA) was found in mammalian cells more than 25 years ago [5]. Since then, several studies have proposed it as a universal signaling molecule [6, 7]. Structurally, ABA is very similar to the TDZs. Indeed, ABA can improve glucose tolerance [8], reduce the level of TNFα, and decrease adipocyte cell size in an in vivo model of obesity induced by HFD [9]. Moreover, in human and murine pancreatic cell lines (RIN-m and INS-2 cells), ABA can increase glucose-stimulated insulin secretion [10]. This effect can be repressed using pertussis toxin and PKA inhibitors [11]. Dietary ABA also stimulates granulocyte function and macrophage infiltration in the adipose tissue [12]. In mammalian cells, the lanthionine synthetase C-like protein 2 (LANCL2) shows high homology with the ABA receptor in plants, the *Arabidopsis* GCR2. Silencing the expression of endogenous LANCL2 in granulocyte cells can abrogate ABA induction of Ca^2+^ responses, whereas overexpression of LANCL2 enhances the ABA-mediated effects [13]. Because of its role in the mediation of ABA effects, LANCL-2 has been proposed as a therapeutic target for the treatment of inflammatory diseases and diabetes [14].

Furthermore, ABA shows some molecular structural similarities to retinoic acid (RA). RA has beneficial cognitive effects, rectifying memory deficits in rodent models of Alzheimer disease. However, the clinical and animal model data show an association between RA administration and the symptoms of depression [15]. Chronic treatment with ABA has beneficial antidepressant effects demonstrated by increased sucrose intake, increased swimming in the forced swim test, and reduced expression of CRH and RARα mRNA in the rat hypothalamus in control rats, with no reported side effects [16]. Moreover, preliminary data show an improved spatial memory in rats treated with ABA [17]. On the basis of these data, we hypothesized that as ABA can modulate peripheral insulin resistance and immune response, it might also exert similar action centrally. Thus, it this work aims to show whether dietary ABA could restore cognitive deficits resulting from a high fat diet (HFD) induced neuroinflammation. HFD elevates the levels of neuroinflammation markers in the brain [18] and it might constitute a link between obesity and degenerative disorders via insulin resistance [19]. Furthermore, HFD has been shown to induce memory loss through elevation of inflammatory markers in hippocampus [20].

We administered ABA (20 mg/L) in the drinking water to rats on either HFD or standard diet (SD). After eleven weeks of treatment, we compared the behavior of the four groups using two memory paradigms: the novel object recognition (NOR) and the T-maze. In addition, we measured ABA levels in the blood and cerebellum of all four groups using HPLC. We analyzed the microglia proliferation using immunohistochemistry. We demonstrated that the ABA administered in drinking water improved glucose tolerance and cognitive performance and decreased the levels of inflammatory markers in the hypothalamic areas. Our results confirm a therapeutic potential of this phytohormone in the peripheral metabolic alterations. The data also strongly suggest the potential beneficial effects of ABA in disorders of neuroinflammatory etiology, which has not been demonstrated before.

## Methods

**Fig. 1:**
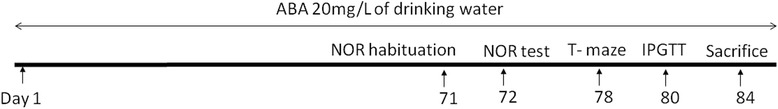
Experiment design. Behavioral tests started in the 11^th^ week, and the animals were sacrificed in the 11^th^ and 12^th^ week

The aim of the experiment is to evaluate the central effects of the phytohormone, ABA in a model of neuroinflammation elicited by HFD, during 12-week treatment. Behavioral tests started on the 11^th^ week, and sacrifices were carried out in the 12^th^ week of ABA and diet treatment (Fig. 1).

### Animals and diet

Eight-week-old male Wistar rats were obtained from the Janvier Labs (Saint-Berthevin, France) and kept at the animal facility of the University Jaume I. The procedures followed the directive 86/609/EEC of the European Community on the protection of animals used for experimental and other scientific purposes. The experiments were approved by the Ethics Committee of the University Jaume I. The animals were maintained on a 12 h:12 h light–dark cycle and housed in pairs to reduce stress due to social isolation. Rats were divided randomly into four experimental groups: SD, control animals fed the standard rodent diet (Ssniff, Soest, Germany); SD-ABA, animals fed standard diet supplemented with ABA (Fernandez-Rapado, Spain) in the drinking water (20 mg/L); HFD, animals fed a high-fat diet (5736 kcal/kg, Ssniff) (Table 1); and HFD-ABA, animals fed high fat diet and ABA in drinking water (20 mg/L). We based our estimations in previous papers where ABA had been administered in the food pellet. Considering the daily food (20 g/day) and water average intake (50 mL/day), we estimated the amount of ABA per mL of drinking water, in order to doses in the same range as previous papers (100 mg/Kg) [21]. In Guri study, the period of ABA treatment was shorter (36 days) than our study (12 weeks), therefore we considered that the ABA administration could be lowered (1 mg/day) to achieve optimal chronic effects. The four groups were fed *ad libitum* for 12 weeks. The HFD diet induces inflammatory effects in the peripheral tissues [21] and the brain [22]. The body weight and food and water intake were monitored twice a week per cage.

**Table 1:** Composition of high fat diet

Crude Nutrients	%	Additives	per kg
Crude protein	24.4	Vitamin A (IU)	15000
Crude fat	34.6	Vitamin D_3_ (IU)	1500
Crude fibre	6.0	Vitamin E (mg)	150
Crude ash	5.5	Vitamin K_3_ (mg)	20
Starch	0.1	Vitamin C (mg)	30
Sugar	9.4	Copper (mg)	12
Fat	60 kJ%		
Energy	21.6 MJ (or kcal) ME/kg		

### Novel object recognition (NOR)

Training chamber consisted of a black wooden box (81 × 61 × 52.3 cm). Rats were habituated to the testing environment for 30 min on two consecutive days. On the first day, the rats spent a 10-min habituation period in the experimental box, without objects. On the second day, each rat was placed in the box for the *familiarization* phase and allowed to explore two identical objects (heavy metallic jars). One group (easy task group) was left for 10 min, and the second group (difficult task group) was left to explore for 3 min. All the rats were then returned to their home cages for a 1-h intertrial interval. The box and objects were cleaned with a 30 % ethanol solution. In the *test* phase, the rats were returned to the box and allowed to explore a familiar and novel object for further 5 min (easy task group) or 3 min (difficult task group) [23]. Familiar and novel objects were alternated between left and right to prevent location predisposition. Both trial and test phases were recorded using a video tracking system (Smart 2.5.19, Panlab, Barcelona, Spain) for subsequent behavioral analysis. Exploration was defined as time spent sniffing within 1–4 cm of the object or touching it, always with the head oriented towards the object. Climbing over the object or running around it was not considered exploration.

### T-maze

The T-maze was a three-arm maze; one arm (119.3 × 18.2 cm) was longer than the other two, which were identical (21.1 × 34.5 cm); the entire maze was placed above the floor. The longer arm was chosen as the start arm. The rats were habituated to the behavior room for 30 min. On the test day, the animals were allowed to explore the maze for 5 min, with access to two of the three arms (the home or start arm and the familiar arm). The rat was then returned to its home cage for a 2-h intertrial interval (difficult task group) or 90-min intertrial (easy task group), during which the maze was cleaned with 30 % ethanol. The rat was then placed back in the maze; this time the animal had access to all arms for 5 min [24]. The number of entries to the novel arm and the time rat spent in each arm was recorded using a video tracking system (Smart 2.5.19).

### Glucose tolerance test

Rats were fasted overnight, and a drop of blood was taken from the tail before (t = 0) and 30 min (t = 30) and 120 min (t = 120) after glucose administration (2 g/kg). Plasma glucose was measured using Glucomen LX Plus glucometer.

### HPLC ABA measurements

ABA was analyzed in all four groups using LC/ESI-MSMS, essentially as described in [25], with slight modifications. Briefly, 1 g of frozen tissue was extracted in ultrapure water using a tissue homogenizer (Ultra-Turrax, Ika-Werke, Staufen, Germany) after spiking with 100 ng of *d*_*6*_-ABA. After extraction and centrifugation, pH of the supernatant was adjusted to 3.0, and it was partitioned twice against diethyl-ether (Panreac, Barcelona, Spain). The organic layers were combined and evaporated in a centrifuge vacuum evaporator (Jouan, Saint-Herblain, France). The dry residue was then resuspended in water–methanol (9:1) solution, filtered and injected into a UPLC™ Acquity system (Waters, Milford, MA, USA). The analyte was then separated on a reversed phase UPLC C18 column (Nucleodur C18, 1.8 μm, 50 × 2.0 mm, Macherey-Nagel, Barcelona, Spain). The solvents were methanol and water supplemented with 0.01 % acetic acid, applied at a flow rate of 300 μL min^−1^. ABA was quantified with a Quattro LC triple quadrupole mass spectrometer (Micromass, Waters, Manchester, UK) connected online to the output of the column through an orthogonal Z-spray electrospray ion source. Quantitation of this hormone was achieved by external calibration with known amounts of standards.

### Immunocytochemistry (ICC)

Rats were anesthetized with an overdose of pentobarbital (120 mg/kg Eutanax, Fatro, Barcelona, Spain) and transcardially perfused with saline (0.9 %) followed by 4 % paraformaldehyde (PFA) fixative in 0.1 M phosphate buffer, pH 7.4. After the perfusion, the brains were removed from the skulls and postfixed overnight at 4 °C in PFA. Then, the brains were immersed in 30 % sucrose for 48 h for cryoprotection. Sliding Microtome Leica SM2010R (Leica Microsystems, Heidelberg, Germany) was used to obtain 40-μm-thick coronal frozen sections. The brains were cut in rostrocaudal direction; six series of slices were collected from each brain and stored at −20 °C for analysis.

For Iba1 staining, we used a goat anti-Iba1 (Abcam, Cambridge, UK). Briefly, free-floating sections were rinsed twice in 0.05 M Tris-buffered saline (TBS, pH 8.0) and once in TBS with 0.2 % Triton X-100, at room temperature. Sections were incubated in 4 % normal donkey serum for 1 h to reduce nonspecific labeling. Afterward, the sections were incubated in the primary antibody solution diluted 1:500 in 0.01 M phosphate buffered saline (PBS) containing 2 % normal donkey serum, TBS with 0.2 % Triton X-100, and 2 % bovine serum albumin for 24 h at room temperature. After washing off the excess of the primary antibody, the sections were incubated in biotinylated donkey anti-goat secondary antibody (Jackson) (1:200 in TBS, 0.2 % Triton X-100). Two hours later, the sections were transferred to the avidin–biotin–horseradish peroxidase complex solution (Standard Elite ABC kit, Vector Laboratories Burlingame, CA USA) for 90 min, followed by two rinses with TBS with 0.2 % Triton X-100, and two more with 0.05 M Tris/HCl pH 8.0. Then, the sections were processed in 0.05 M Tris/HCl pH 8.0 containing 3.125 mg of DAB and 2 μL of H_2_O_2_ for 15–20 min. The reaction was stopped by adding an excess of 0.05 M Tris/HCl pH 8.0, followed by several rinses in PBS. Finally, the sections were mounted onto gelatin-coated slides, air-dried, dehydrated in alcohol, cleared in xylene, and coverslipped with DPX mounting medium.

### RNA extraction and RT-PCR

Total RNA was extracted from the hypothalamus (*n* = 4–6 per group) using RNeasy Lipid Tissue Mini Kit (Qiagen, Valencia, CA, USA) according to the manufacturer’s protocol. The RNA samples were resuspended in 50 μL of nuclease-free water. RNA concentration and quantification of total RNA was performed using Thermo Scientific NanoDrop 2000c, with the OD260/OD280. Genomic DNA was removed using DNase I, RNase-free (Life Technologies, USA), for 30 min at 37 °C. The reaction was stopped by addition of 1 μl of EDTA for 10 min at 65 °C. The first strand cDNA was synthesized using the PrimeScript™ RT Reagent Kit (Perfect Real Time) (Takara Bio Inc., Shiga Japan). For each reaction, 1 μg of RNA was used for reverse transcription, in a mixture of 4 μL of 5 × PrimeScript Buffer; 1 μl of PrimeScript RT, 1 μL of Oligo dT Primer (50 μM), and 1 μL of random primer (100 μM). Enzyme mix was adjusted to a final volume of 20 μL at room temperature. The mixture was incubated at 37 °C for 15 min and heated at 85 °C for 15 min to terminate the reaction. The cDNA was subsequently stored at −20 °C. RT-PCR was performed in a volume of 10 μL with 5 μL of Maxima SYBR Green/ROX qPCR Master Mix (2X) (Applied Biosystems Life Technologies, Carlsbad, CA, USA), 1 μL of primer and 1 μL of cDNA. All PCR reactions were performed under the following conditions: initial cycle at 98 °C for 10 min followed by 40 cycles at 98 °C for 10 s, 60 °C for 10 s, and 72 °C for 20s. Gene expression in the hypothalamus and hippocampus was quantified using a StepOnePlus Real-Time PCR system (Applied Biosystems Life Technologies). The RT-PCR primers for TNFα were Forward 5′GACCCTCACACTCAGATCATCTTCT3′ and reverse 5′TGCTACGACGTGGGCTACG3′. Each sample was tested in triplicate. Data were analyzed using the comparative critical threshold method, with the amount of target gene normalized to the housekeeping gene ß-actin. Relative gene expression was calculated using 2 -^ΔΔCt^ relative to control.

## Results

**Fig. 2:**
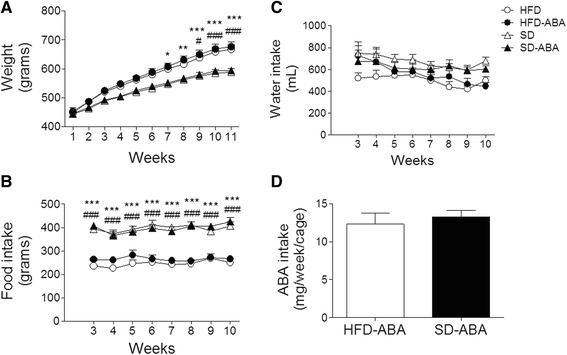
Body weight in grams (**a**), food intake (**b**), water intake (**c**) and ABA weekly comsumption per cage (**d**) of rats fed high-fat diet (HFD, white circles); HFD with ABA (HFD-ABA, black circles); standard diet (SD, white triangles), and SD-ABA (black triangles) for 10–11 weeks; *n* = 16 per group. Values are mean ± SEM. #, *p* < 0.05; ##, *p* < 0.01 for HFD *vs.* SD. *, *p* < 0.05; **, *p* < 0.01, ***, *p* < 0.001 for HFD-ABA *vs.* SD

Animals were weighed, and water and food consumption was monitored twice a week. Behavioral tests started on the 11^th^ week, and sacrifices were carried out in the 12^th^ week of ABA and diet treatment (Fig. 1). As expected, overnutrition affected the body weight, but ABA administration did not affect the weight gain in either group. The results are presented as the means ± SE (*n* = 16 per group). Control animals fed SD increased their body weight from 444 ± 11.7 g (week 1) to 585 ± 12.0 g (week 10). Similarly, animals on SD supplemented with ABA increased their body weight from 446 ± 10.7 g (week 1) to 593 ± 10.2 g (week 10). This represents an increment of 132 ± 2.4 % and 133 ± 1.5 %, for SD and SD with ABA, respectively. However, animals fed HFD and HFD-ABA increased their body weight from 448 ± 11.7 and 453 ± 12.2 g (week 1) to 659 ± 16.0 and 669 ± 16.2 g (week 10), an increase of 148 ± 3.2 % and 148 ± 2.8 %, respectively (Fig. 2a). Data were analyzed using a two-way ANOVA; time (F_(10,600)_ = 994, *p* < 0.0001) and diet (F_(3,60)_ = 6.08, *p* = 0.001) had a significant effect. Food and water consumption was measured per cage. Food intake data is represented by the mean (g) of food consumed ± SE per cage each week (*n* = 8 per group). Weekly consumption was steady during the 12 weeks treatment, but the diet clearly affected food intake. The data were analyzed using two-way ANOVA (F_(3,28)_ = 75.36 *p* < 0.0001). To determine the ABA intake, we monitored weekly water consumption (Fig. 2c). Based on this information, we calculated ABA intake for both HFD and SD fed animals, and confirmed that both groups had a similar average weekly intake of the hormone, 12.30 ± 1.4 and 13.27 ± 0.8 mg/week/cage, respectively (Fig. 2d).

**Fig. 3:**
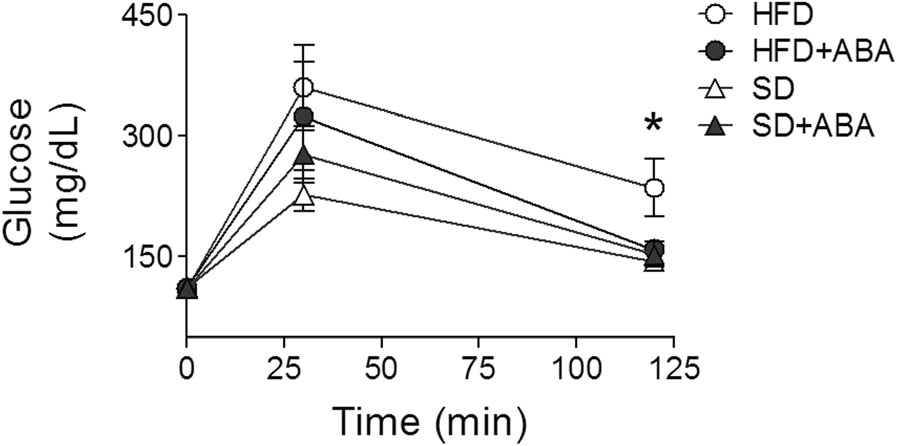
The effect of dietary ABA (20 mg/L drinking water) on blood glucose levels in response to intraperitoneal (IP) glucose tolerance test. After 12 weeks of treatment, animals from the four groups were fasted overnight (12–13 h) and given 2 mg/kg of glucose by IP injection. Blood drops were taken from the tail, and plasma glucose concentration measured at the indicated times. Data are presented as the mean ± SEM. White circles represent rats fed HFD; black circles, HFD-ABA; white triangles, SD; and black triangles, SD-ABA. Data points at 120 min were analyzed using one-way ANOVA followed by post-hoc Newman–Keuls test. *, *p* < 0.05

It has been reported that dietary ABA given in the food pellets (100 mg/kg food) can improve glucose tolerance [21]. To confirm that, in our model, ABA (20 mg/L of drinking water) improved the glucose homeostasis, we performed a glucose tolerance test by intraperitoneal injection of glucose (2 mg/kg) in fasted animals. After 11 weeks of treatment, the animals fasted overnight (12–13 h). The basal glucose levels were similar in all groups; 30 min after glucose injection, blood glucose levels increased. No significant differences were observed between the groups. However, two hours after injection, glucose levels in HFD group remained higher (235.4 ± 36 mg/dL) than in HFD-ABA group (158.9 ± 11.23 mg/dL). In the latter group, the glucose levels were similar to those in control groups, SD (143.9 ± 10.24 mg/dL) and SD-ABA (152.6 ± 15.52 mg/dL). The data were analyzed using one-way ANOVA, with Newman–Keuls post-hoc test (*p* < 0.05) (Fig. 3).

**Fig. 4:**
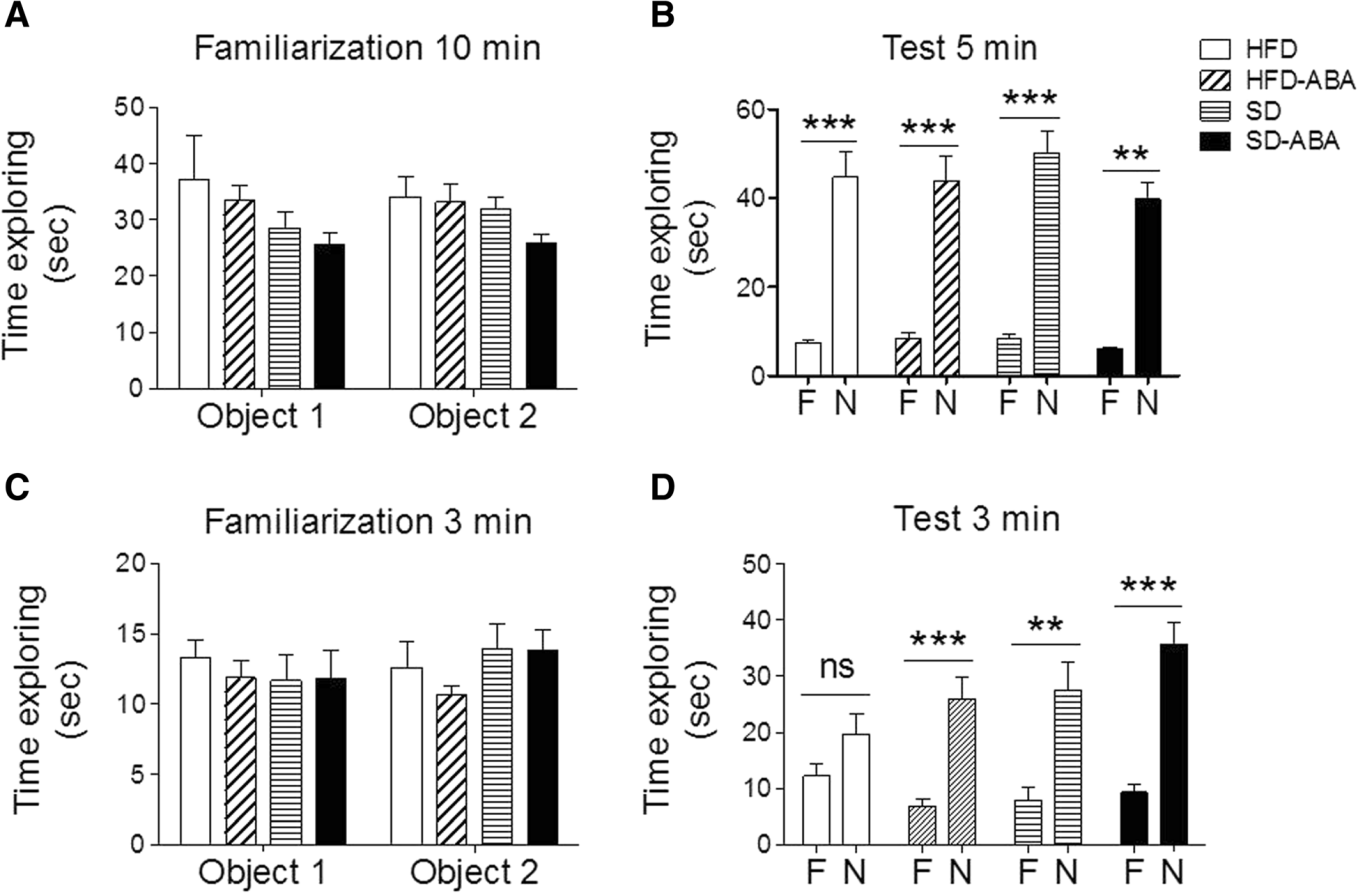
Behavioral data for rats on a standard diet (horizontal lines, SD), high-fat diet (white columns, HFD), and supplemented with ABA in drinking water (20 mg/mL), HFD-ABA (diagonal lines) and SD-ABA (black columns) (*n* = 8). Novel Object Recognition data: time exploring two similar objects during familiarization phase (**a**, **c**) and time exploring familiar and novel object during test phase (**b**, **d**). Data is presented as mean ± SEM and analyzed using paired Student *t*-test, familiar *vs.* novel. **p < 0.01; *** *p* < 0.001; ns, non-significant

HFD can induce memory impairments in rodents [26] and humans [27]. To examine the effect of chronic ABA administration on cognitive performance, the animals were subjected to two behavioral paradigms that evaluate the memory in rodents, NOR and T-maze test. NOR exploits the innate exploratory preference of novel objects exhibited by rodents. This paradigm examines the capability of the animal to remember a familiar object when presented with a new one. We observed no significant differences in the time spent exploring the identical objects during familiarization phase (Fig. 4a and c). During the test, all four groups spent much more time exploring the novel object than the familiar one, and neither the diet nor ABA treatment changed these parameters (Fig. 4b). However, we observed differences when both the familiarization time and test time were reduced to 3 min. In the HFD group, the times for familiar and novel object did not differ significantly, suggesting impairment in remembering the familiar object. HFD-ABA animals behaved in the same way as the control groups (SD and SD-ABA), indicating that ABA could abrogate the HFD-induced impairment (Fig. 4d).

**Fig. 5:**
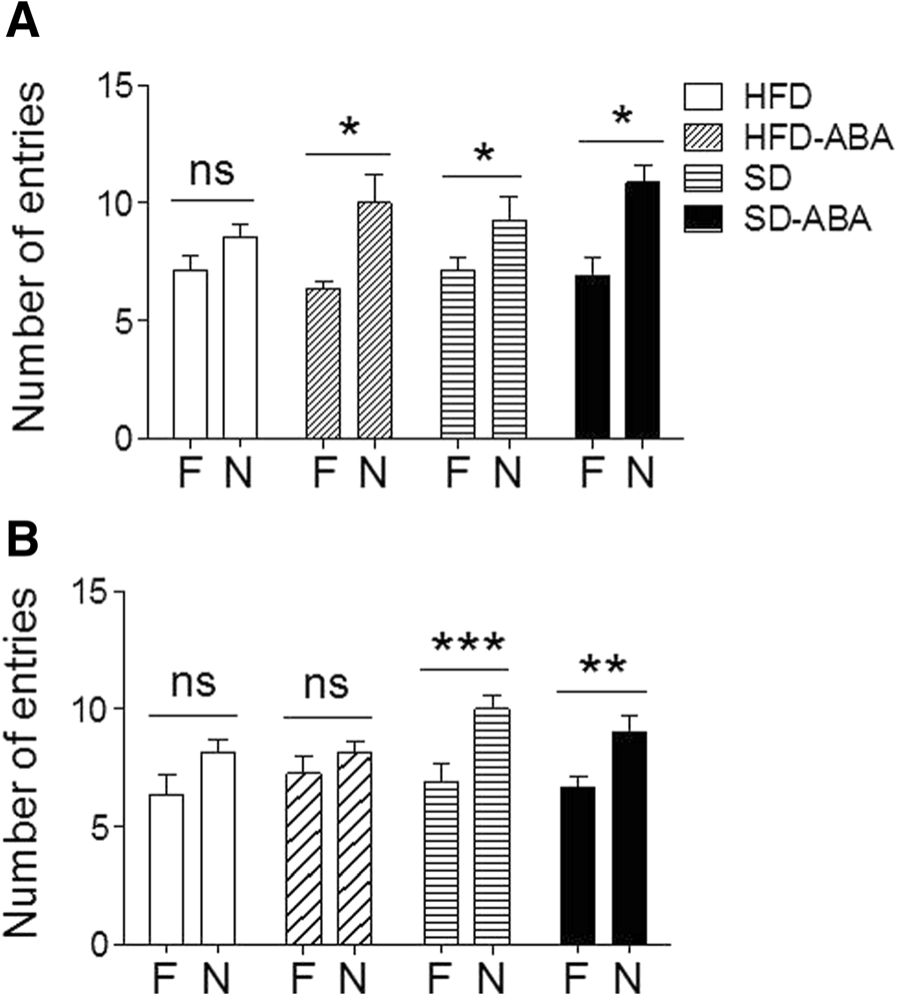
Behavioral data for rats on a standard diet (horizontal lines, SD), high-fat diet (white columns, HFD), and supplemented with ABA in drinking water (20 mg/mL), HFD-ABA (diagonal lines) and SD-ABA (black columns) (*n* = 8). T-maze data: number of entries to novel and familiar arm after 90 min intertrial interval (**a**) and number of entries to novel and familiar arm after 2 h intertrial interval (**b**). Data is presented as mean ± SEM (*n* = 8) and analyzed using paired Student *t*-test, familiar *vs.* novel. *p < 0.05; **p < 0.01; *** *p* < 0.001; ns, non-significant

In the T-maze test, we recorded the number of entries to both maze arms, one of which was familiar and the other was novel because it had been closed during the habituation. We observed that rats fed SD, SD-ABA, and HFD-ABA had a significantly higher number of entries to the novel arm than to the familiar one. This was not the case for the animals fed HFD only, suggesting that this diet might impair the memory of the familiar arm (Fig. 5a). The data were analyzed using unpaired Student’s *t*-test, comparing familiar and novel arm; *p < 0.05 < **p < 0.01. Interestingly, when the test was performed with a longer inter-trial time between familiarization and test phases, which may be a more difficult working memory task, HFD-ABA did not rescue the alternation behavior shown by the HFD group. We found that the difference between the number of entries to the two arms was no longer significant for HFD-ABA animals, in similarity with HFD group. Both SD and SD-ABA group maintained a significant difference in the exploratory behavior, entering the novel arm more often (Fig. 5b).

ABA levels in the blood and brain (cerebellum) were measured using HPLC. We detected variable amounts of circulating ABA in treated animals. However, we were unable to detect ABA under the same conditions in untreated animals, confirming that we, indeed, were observing exogenous ABA (Table 2). It has been reported that after intraperitoneal injections, ABA is widely distributed in the brain [17]. To ascertain that ABA crosses the blood–brain barrier in our model, we measured the ABA levels in the cerebellum as a sentinel. In similarity with the serum examination, ABA was only detected in the brains of treated animals (Table 2). Interestingly, only a very small proportion of circulating ABA crosses the blood–brain barrier. Thus, we confirmed that our method of ABA administration in drinking water was appropriate for measuring potential peripheral effects; the compound could cross the brain–blood barrier and might be responsible for the observed behavioral changes.

**Table 2:** ABA quantification by HPLC. Cerebellum (A) and blood (B) were extracted from all four groups of treated animals

Cerebellum		Serum	
Diet group	ABA detected (ng/gr tissue)	Diet group	ABA detected (ng/gr plasma)
HFD	N.D	HFD	N.D
HFD-ABA	1.510 ± 0.030	HFD-ABA	196.8 ± 82.57
SD	N.D	SD	N.D
SD-ABA	2.980 ± 0.100	SD-ABA	47.95 ± 13.80

ND not detected

ABA was only detected in animals that had received exogenous administration of ABA in the drinking water (20 mg/L)

**Fig. 6:**
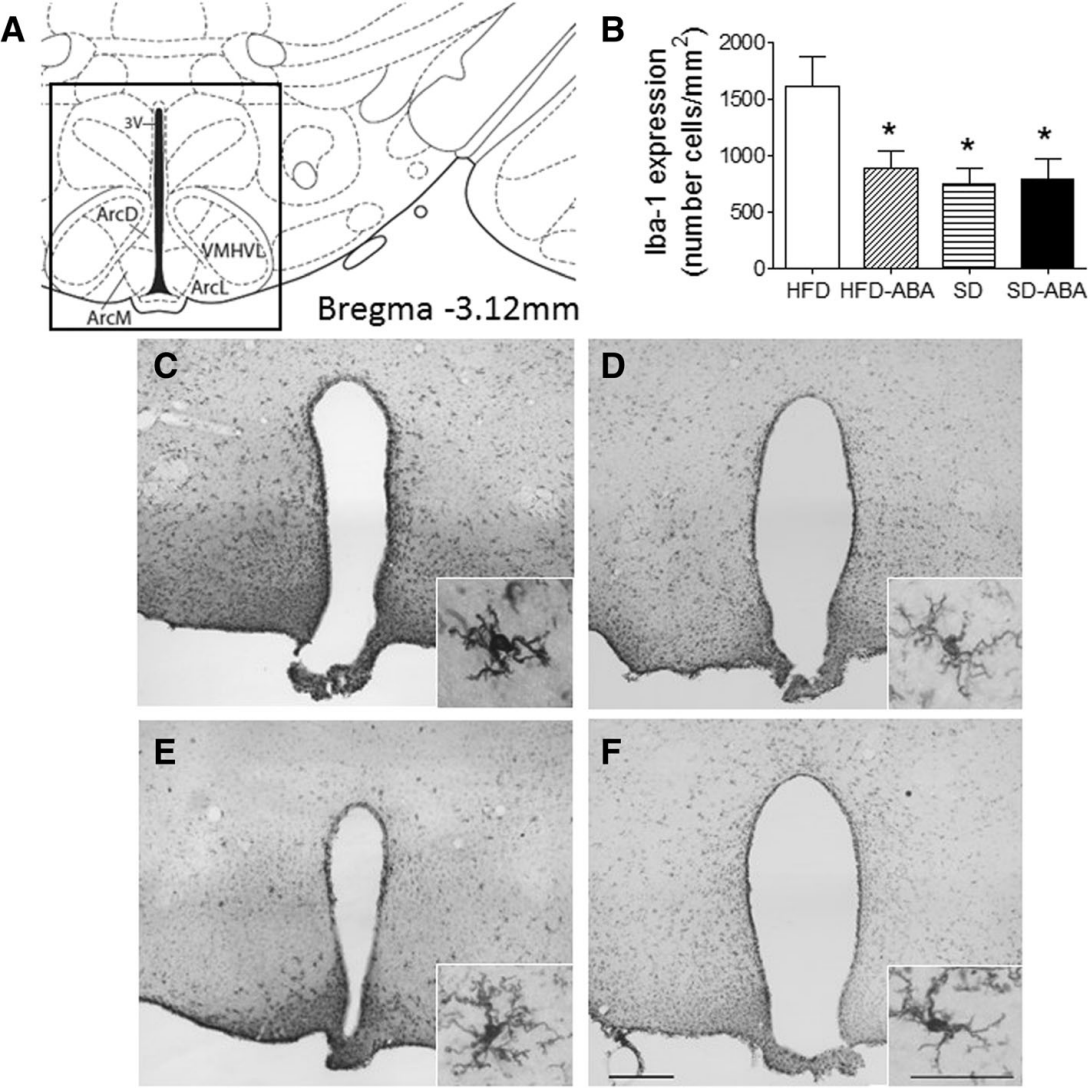
Within the hypothalamus, ABA curtails the increase in microglia staining induced by high-fat diet (HFD). Map showing the location of the analyzed hypothalamus (**a**). Anterior (bregma −1.80 mm) and medial (bregma −3.12 mm) hypothalamus were used for quantification (**b**). HFD fed rats (**c**); HFD-ABA (**d**); standard diet, SD (**e**); and SD-ABA (**f**). Calibration bar, 200 μm (**c**–**f**). Data is represented as the mean ± SEM of density of cells per mm^2^ (*n* = 6). Data were analyzed using one-way ANOVA followed by post hoc Newman–Keuls test; *, *p* < 0.05 *vs.* HFD. The reactive microglia are shown in the insets at high magnification. Calibration bar, 50 μm

Next, we measured the levels of inflammatory markers in the brain. We used immunohistochemistry to detect the microglia-specific cytoplasmic marker Iba1 [28] in the hypothalamus (Fig. 6a). We observed that the number of microglial cells increased significantly in rat hypothalamus of HFD-fed rats (1613 ± 260.5 cells/mm^2^) in comparison with SD-fed controls (754 ± 135.2 cells/mm^2^). The ABA treatment reduced this effect in HFD animals (888 ± 158.8 cells/mm^2^) but did not affect SD-fed animals (794 ± 178.4 cells/mm^2^) (Fig. 6b; one-way ANOVA, *p* = 0.0135). Representative images of microglia in the hypothalamus are shown in Fig. 6 (HFD, Fig. 6c; HFD-ABA, Fig. 6d; SD, Fig. 6e; and SD-ABA, Fig. 6f). In HFD group, apart from the increase in the number of Iba1-positive cells, the microglia cells were less ramified and more amoeboid than in controls, indicating an activated state (insets in images).

**Fig. 7:**
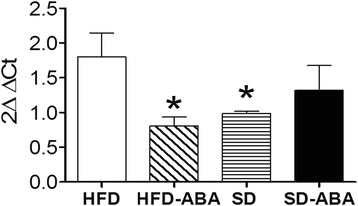
Cytokine mRNAs levels in the hypothalamus: proinflammatory cytokine TNFα in rats fed high fat diet (HFD, white columns); HFD supplemented with ABA in drinking water (20 mg/L) (HFD-ABA, diagonal lines); standard diet (SD, horizontal lines); and SD supplemented with ABA (SD-ABA, black columns). Data are presented as mean ± SEM (*n* = 4–6 rats) and analyzed using one-way ANOVA followed by Newman–Keuls Multiple Comparison Test; *, *p* < 0.05 *vs.* HFD

Next, we examined the levels of inflammatory and anti-inflammatory cytokines in the hypothalamus. The hypothalamus was dissected from all groups, and total RNA was isolated. Quantitative PCR was carried out to determine the expression levels of different cytokines. We observed that TNF-α levels increased in the hypothalamus of HFD-fed rats in comparison with controls, confirming the existing reports [29, 30]. ABA administration restored TNFα levels to control values (Fig. 7). On the other hand, we found no significant differences in hippocampus, under the same conditions in all four groups (data not shown).

## Discussion

Excessive energy intake is a major cause of obesity, which is recognized as one of the greatest threats to human health in the Western societies. Malfunction of hypothalamic neurons appears to underlie the pathology of metabolic diseases [31]. Even though the relationship between obesity (excess body fat) and neurodegeneration is highly complex, many sources link obesity and high fat consumption with certain neurodegenerative processes [19]. Growing evidence demonstrates that HFD induces insulin resistance, immunological and synaptic alterations in different brain areas [32, 33], and cognitive impairment [34].

In animal obesity models, the phytohormone ABA, related structurally to insulin sensitizers (TDZs) and memory-improvement molecules (such as retinoic acid), ameliorates the symptoms of type 2 diabetes. It targets PPAR-γ in a manner similar to the TDZ class of antidiabetic drugs [21, 35]. In this study, we demonstrate, for the first time, that ABA can counteract some of the neurological effects (cognitive impairment and neuroinflammation markers) induced by HFD. We, in this study, and others have found that ABA can cross the blood brain barrier, but whether the improvement on cognitive functions is caused by direct action of ABA in brain, remains elusive.

Our protocol was designed to avoid an increase in behavioral anxiety. ABA was administered in the drinking water (20 mg/L) and not in food pellets since rats fed on an HFD tend to eat less than controls [36]. The dose was based on previous studies reporting dietary administration of ABA [21]. The vehicle used to dissolve ABA was added to water for SD and HFD groups to match any possible taste alterations. Some studies have used intraperitoneal injections (IP), which guarantee exact dosing but are highly anxiogenic. Another innovation in our study was housing the animals in pairs to reduce stress due to isolation [37]. We considered these issues important since stress can affect feeding and cognitive behavior in many indirect ways [38]. Therefore, reducing the stress levels (by avoiding isolation and daily injections) was desirable. To examine the intake of ABA, we examined the average weekly water consumption in groups SD-ABA and HFD-ABA. Daily ABA intake was approximately 1.5–1.8 mg/kg. During the 12-week period, the intake average was uniform in all groups. To validate our strategy as an effective way of drug administration, we carried out a glucose tolerance test in all four groups. We confirmed that ABA improves glucose tolerance, reducing blood glucose levels to the normoglycemic range two hours after glucose injection.

The effect of specific diets on cognitive performance in rodents has been investigated using different behavioral paradigms. Diets with increased sugar and fat content cause impairment in spatial and working memory [39–41] in both rats and mice. The link between obesity and inflammation of central nervous system is widely accepted; the hypothalamus is the area where inflammatory markers are most evident. Neuroinflammation underlies cognitive impairment [1]. However, some reports have suggested that ABA improves spatial memory [16]. ABA has anti-inflammatory and insulin-sensitizing properties, and there is some evidence suggesting that ABA is a PPARγ agonist [42, 43]. Some reports have shown that PPARγ agonism in the central nervous system ameliorates cognitive function [44, 45]. Therefore, we hypothesized that ABA would alleviate the HFD-induced neuroinflammation and cognitive defects. To follow up this assumption, we examined the performance of the rats in two simple memory tests, NOR and T-maze. In contrast to other reports, ABA did not significantly affect the animals fed a control diet. This discrepancy might have been caused by differences in animal handling. As mentioned above, we housed the animals in pairs to reduce the isolation anxiety and ABA was administered in the drinking water, reducing the IP injection-induced stress. Under these conditions, the reduced anxiety levels might have a positive effect on test performance. In the studies showing antidepressant-like effects of ABA, the compound has been administered daily using an IP injection [17]. We believe that in our model, the effect of ABA is independent of its antidepressant activity.

In an NOR working-memory test, all groups, including the HFD group, were able to discriminate between the novel and the familiar object. However, when the time spent in familiarization and test phases was shortened, thus reducing/hurting the acquisition phase, HFD-fed animals spent similar time exploring the novel and the familiar object. This indicated that they no longer remembered the familiar object. This behavior was efficiently counteracted by ABA treatment. ABA-treated animals behaved like the members of other groups, spending significantly more time exploring the novel object.

The alternation test (the T-maze test) is considered a reliable test to evaluate spatial working memory in rodents [46, 47]. Using this paradigm, we found that HFD could impair alternation; the animals had difficulties in remembering the previously visited arms. Chronic treatment with ABA can restore this capability. However, when the intervals between the tests were increased, making the task harder, HFD-ABA-fed animals no longer alternated, behaving like the animals in the HFD group. This indicated that they had lost the ability to discriminate between the familiar and novel arms. We would not attribute these differences to ABA metabolism, not only ABA is administered chronically, but during the intertrial interval rats have access to water and food. The peripheral anti-inflammatory effects ABA are well known. However, to the best of our knowledge, there is no data on the effect of ABA on the cerebral immune response in vivo. Moreover, in vitro data are controversial; some studies show that ABA activates the microglia [48], whereas others report no effect [49]. Among the different types of glia, the resident macrophages in the brain, microglia, are responsible for cell debris clearance and release of cytokines that recruit other immunoresponsive cells to the central nervous system [50]. In the metabolic syndrome induced by HFD, microglia in the hypothalamus adopt a proinflammatory state, which is linked to an abnormal increase in the production of proinflammatory cytokines. These might be toxic to the neurons [51, 52]. In this study, we investigated the microglial expression in the hypothalamus in the four study groups. We examined the immunoreactivity of ionized calcium-binding adaptor molecule 1 (Iba1-ir), a cell marker upregulated during microglial activation [28]. In contrast to other studies of ABA effect on microglia in vitro, reporting either activation [46] or no effect [49], we demonstrated that in vivo, ABA reduces the number of Iba1-positive cells. This finding suggests an important practical application; the microglia hyperactivation has been found in a number of alterations including traumatic brain injury [53], cerebrovascular accidents [54], neurodegenerative disorders [55, 56], epilepsy [57], schizophrenia [58], and depression [59]. In addition, we analyzed Iba-1 expression in dorsal hippocampus, but, HFD did not increase microglia proliferation compared to control groups (data not shown).

Further to test the anti-inflammatory effects of ABA in the central nervous system, we measured the hypothalamic and hippocampal cytokine levels. The activated microglia synthesize and secrete proinflammatory cytokines, such as TNFα [60]. We observed an increase in TNFα levels in the hypothalamus of HFD-fed rats. ABA treatment rescued this effect, lowering TNFα to control levels. Contrary to other studies where inflammation markers have been found to increase in hippocampus, we found no significant difference in inflammatory markers TNF-α with HFD treatment compared to control groups. We postulate that not only hippocampal neuroinflammation underlies cognitive impairments.

## Conclusion

In summary, we found that chronic treatment with ABA reduced HFD-induced microglia activation in hypothalamus, as revealed by Iba-1 staining. Also, TNF-α levels were altered in hypothalamus of HFD-fed animals, and these changes were counteracted by ABA administration. Moreover, ABA ameliorated the HFD-induced impairments in cognitive performance. We found no changes in neuroinflammatory markers in hypothalamus. In this study, we show that hypothalamic inflammation can correlate with mild alterations in cognitive performance. Finally, based on our results, we conclude that ABA has a protective effect in neuroinflammation conditions, lowering microglia and TNFα levels and restoring cognitive function. Given the fact that ABA can cross the blood brain barrier; we postulate that this molecule could have a potential therapeutic value in the treatment of diseases of neuro-inflammatory etiology.

## Abbreviations

ABA: Abscisic acid
ANOVA: Analysis of variance
CRH: Corticotropin releasing hormone
GCR: G-protein coupled receptor
HFD: High-fat diet
HPLC: High pressure liquid chromatography
ICC: Immunocytochemistry
LANCL2: Lanthionine synthetase C-like protein 2
NOR: Novel object recognition
PBS: Phosphate buffered saline
PFA: Paraformaldehyde
PPARγ: Peroxisome proliferator-activated receptor gamma
RA: Retinoic acid receptor
RAR: Retinoic acid
RT-PCR: Real time-polymerase chain reaction
SD: Standard diet
TBS: Tris-buffered saline
TDZ: Thiazolidinediones
TNF-α: Tumor necrosis factor
